# Assessment of risk factors and molecular biomarkers in children with supernumerary teeth: a single-center study

**DOI:** 10.1186/s12903-022-02151-z

**Published:** 2022-04-09

**Authors:** Dalia M. Talaat, Ibrahim Y. Hachim, Marwa M. Afifi, Iman M. Talaat, Mona A. ElKateb

**Affiliations:** 1 Pediatric Dentistry and Dental Public Health Department, Faculty of Dentistry, Alexandria University, Alexandria, Egypt; 2 Clinical Sciences Department, College of Medicine, University of Sharjah, Sharjah, UAE; 3 Department of Oral Pathology, Faculty of Dentistry, Alexandria University, Alexandria, Egypt; 4 Laboratory of Cancer Biology and Genetics, Center for Cancer Research, National Cancer Institute, Bethesda, MD USA; 5 Pathology Department, Faculty of Medicine, Alexandria University, Alexandria, Egypt; 6 Department of Preventive Dental Sciences, College of Dentistry, Princess Nourah Bint Abdulrahman University, Riyadh, Saudi Arabia

**Keywords:** Supernumerary teeth, Environmental factors, Molecular biomarkers

## Abstract

**Background:**

Supernumerary teeth are considered one of the commonly observed dental anomalies in children. Several theories have been proposed to explain the presence of supernumerary teeth, including environmental and genetic factors. This study aimed to identify the different risk factors and molecular biomarkers in patients presented with supernumerary teeth.

**Methods:**

This case–control study included 240 children, 6 to 12-year-old. They were divided into a test group (n = 120 children presented with supernumerary teeth) and a control group (n = 120 children with no supernumerary teeth). Questionnaires were distributed to assess demographics and exposure to several environmental factors. Ten extracted supernumerary teeth from the test group were processed for histopathological analysis.

**Results:**

Male gender, dental history of severe oral infection or medical history of chemotherapy treatment, previous history of taking medication or illness during pregnancy, family history of neoplastic disorders, use of electronic devices, and living beside agricultural fields or industrial areas were found to be statistically significant associated with the risk of supernumerary teeth development. Immunohistochemistry panel revealed that supernumerary teeth showed enhanced expression of wingless (Wnt) and sonic hedgehog (SHH) proteins as well as a reduced expression of adenomatous polyposis coli (APC) protein, denoting molecular derangement in a group of pathways classically believed to be involved in its pathogenesis.

**Conclusions:**

Males were more frequently affected by supernumerary teeth than females. Several risk factors were notably correlated with the existence of supernumerary teeth. Additionally, molecular biomarkers assessment demonstrated a high expression level of pro-tumorigenic proteins such as Wnt and SHH in patients with supernumerary teeth.

## Background

Supernumerary teeth or hyperdontia represent an excessive number of teeth of the normal dentition due to an alteration in odontogenesis. It can affect both the primary and/or the permanent dentition. According to the American Academy of Pediatric Dentistry, the prevalence of supernumeraries in the primary dentition ranges from 0.3 to 0.8%, while it ranges from 0.5 to 2% in the permanent dentition [1]. The presence of supernumerary teeth may produce several complications, including delay or failure of permanent teeth eruption, displacement or rotation of adjacent teeth and wide diastema, as well as crowding and dilacerations of adjacent permanent teeth [2–4]. Pathological problems such as resorption of the adjacent roots, dentigerous cyst and ameloblastoma have also been reported [5].

A number of theories have been postulated to explain the presence of supernumerary teeth, including atavism (evolutionary throwback) [6], tooth germ dichotomy [7] and hyperactivity of the dental lamina [8]. Environmental factors such as trauma, infections, radiation, drugs, and hormonal influences have been suggested as possible insults that might affect tooth formation during the embryologic stages [9].

The heterogeneity played a role in supernumerary teeth etiology. It was observed at the molecular level, which led to a better understanding of the different mechanisms involved in tooth formation and development [6]. Odontogenesis is a complex mechanism that is governed by many genetic pathways, including adenomatous polyposis coli (*APC*), wingless signaling pathway (*Wnt*), sonic hedgehog (*SHH*) and bone morphogenic protein (*BMP*) genes [10]. Some of these developmental genes interplay with one another, leading to up-regulation and/or down-regulation of critical cell cycle control molecules [11]. Although the interplay between the genetic and environmental factors during the odontogenesis process can lead to tooth number anomalies, the exact etiology remains unclear [12].

Additionally, it has been suggested that different biological and environmental factors may affect the development of this condition. Therefore, this study aimed to identify the different risk factors and molecular biomarkers of patients presented with supernumerary teeth.

## Methods

### Study design

This is a case–control study. It was set up and reported according to Strengthening the Reporting of Observational Studies in Epidemiology (STROBE) guidelines [13]. The research protocol was approved by the Research Ethical Committee of the Faculty of Dentistry, Alexandria University (#IRB NO: 00010556-IORG 0008839). Signed informed consent from a parent or legal guardian, as well as children’s assent to participate in the study, were obtained.

### Study sample

This study enrolled patients who attended the Pediatric Dental Clinic of the Pediatric Dentistry and Dental Public Health Department, Faculty of Dentistry, Alexandria University, from January 2021 to June 2021 and underwent panoramic radiographs at their initial visits. Selection criteria included: (1) Egyptian ethnicity; (2) Age 6–12 years; (3) No significant medical comorbidities (e.g., systemic/hereditary diseases, syndromes, or craniofacial malformations); (4) Clinical and/or radiographic evidence of supernumerary teeth; (5) Absence of developmental anomalies of size or shape, and (6) No history of extraction or orthodontic treatment.

One hundred and twenty patients met the selection criteria. Subsequently, another 120 controls were selected in 1:1 matching using the same aforementioned criteria except for the presence of supernumerary teeth.

### Clinical examination

Medical and dental histories were recorded, and an expert pediatric dentist performed clinical examination of all children using a mirror and probe under adequate lighting. Radiographic examination using occlusal films was done to confirm the presence or absence of supernumeraries and other abnormalities. Wherever required, they were supplemented with periapical radiographs [1].

### Questionnaire

Parents of both groups enrolled in this study were asked to fill out a questionnaire. The questionnaire inquired about the patients’ demographic data (age and gender), feeding (breast or bottle feeding), drinking mineral or tap water, medical history (infectious diseases or chemotherapy), previous history of dental trauma to the primary dentition, and exposure to electronics, radiation, or pollution, in addition to parental information (parents’ consanguinity, family members with supernumerary teeth and smoking), and medical history (pregnancy medication or illness, malignancy).

### Validity of questionnaire

#### Content validity

Four dental academics were given a content validation form and asked to rate the degree of relevance of each item on a four-point ordinal scale: (1) not relevant, (2) somewhat relevant, (3) fairly relevant, and (4) highly relevant. The content validity at the item level (CVI-I) was computed by dividing the total number of experts by the number of experts who gave a score of 3 to 4 for each relevant item. A CVI-I score of 0.95 was attained overall, which was deemed appropriate [14].

#### Face validity

To examine face validity, 20 parents participated in the pilot testing of the questionnaire to assess its clarity and whether any questions were confusing. A dichotomous scale was used with the categorical option of “Yes” and “No”, denoting a clear and unclear item, respectively [15]. The responses were then subjected to Cohen's Kappa test, which yielded a score of 0.87, reflecting good agreement. These parents were not included in the study.

### Histopathological and immunohistochemical analysis

In the present research, 80% of the supernumerary teeth were extracted and clinically followed up, while 15% were surgically removed and orthodontically treated when required and 5% were followed without removal. Ten of the extracted supernumerary teeth were stored in saline until processed for histopathological and immunohistochemical analysis. Sections from formalin-fixed paraffin-embedded (FFPE) blocks were prepared according to Kassem et al. [16]; one section was stained with hematoxylin and eosin, and four unstained slides were prepared for immunohistochemistry (IHC). For IHC, FFPE sections were deparaffinized in xylene and rehydrated in graded ethanol concentrations. Heat-mediated antigen retrieval was carried out by boiling the sections in 10 mM citrate buffer (pH 6.0) for 10–20 min, then cooling at room temperature for 20 min. Subsequently, sections were washed with phosphate-buffered saline and blocked with hydrogen peroxide (Thermo Fisher Scientific, Fremont, CA, USA) for 5 min followed by ultraviolet block (Thermo Fisher Scientific) for 5 min at room temperature. Sections were incubated with the following primary antibodies according to the manufacturer’s instructions at ambient room temperature for 60 min: (1) Anti-BMP11 (US Biological Life Sciences, catalogue #: 127,251), (2) Anti-SHH (US Biological Life Sciences, catalogue #: 145,161), (3) Anti-APC (ThermoScientific, catalogue #: RB-927), (4) Anti-Wnt4 antibody (Abcam, catalogue #: ab189037), Anti-Wnt5a antibody (Abcam, catalogue #: ab229200), and Anti-Wnt6 antibody (Abcam, catalogue #: ab150588). This was followed by applying a labeled polymer to cover the sections and incubating for 30 min. Then, 3,3′-diaminobenzidine and chromogen were added and incubated in a dark chamber for 5–10 min. Sections were counterstained with hematoxylin stain. Negative controls (omitting the primary antibody) were included in all the runs to check for non-specific signals and false-positive results.

### Analysis of immunohistochemical staining

The immunoreactivity of the different proteins was quantitatively assessed via ImageJ software by an expert histopathologist. For each specimen, immuno-expression was evaluated in terms of area percentage and cell compartment (cytoplasm, nuclear, and/or cell membrane). Odontoblasts and mesenchymal cells were considered positive regardless of the staining intensity. The number of positive cells was counted in 5 microscopic fields (400× magnification) and expressed as mean area percentage. The staining index (area percentage) was calculated as the percentage of the stained cells relative to the total number of cells over the same area [17]. Accordingly, lack of signals reflects the loss of function of the protective tumour suppressor gene APC*,* while the presence of signals indicates the gain of function of the oncogenic genes, namely BMP, SHH and Wnt [18].

### Statistical analysis

Qualitative variables were presented using frequency and percentage, while quantitative variables were presented as mean ± SD. Data were analyzed by GraphPad Prism Version 8 (Graph Pad Software, San Diego, Ca, USA). Responses to the questionnaire were compared using Pearson’s Chi-Square test or Fisher’s Exact. The level of statistical significance was *P* < 0.05.

## Results

**Fig. 1:**
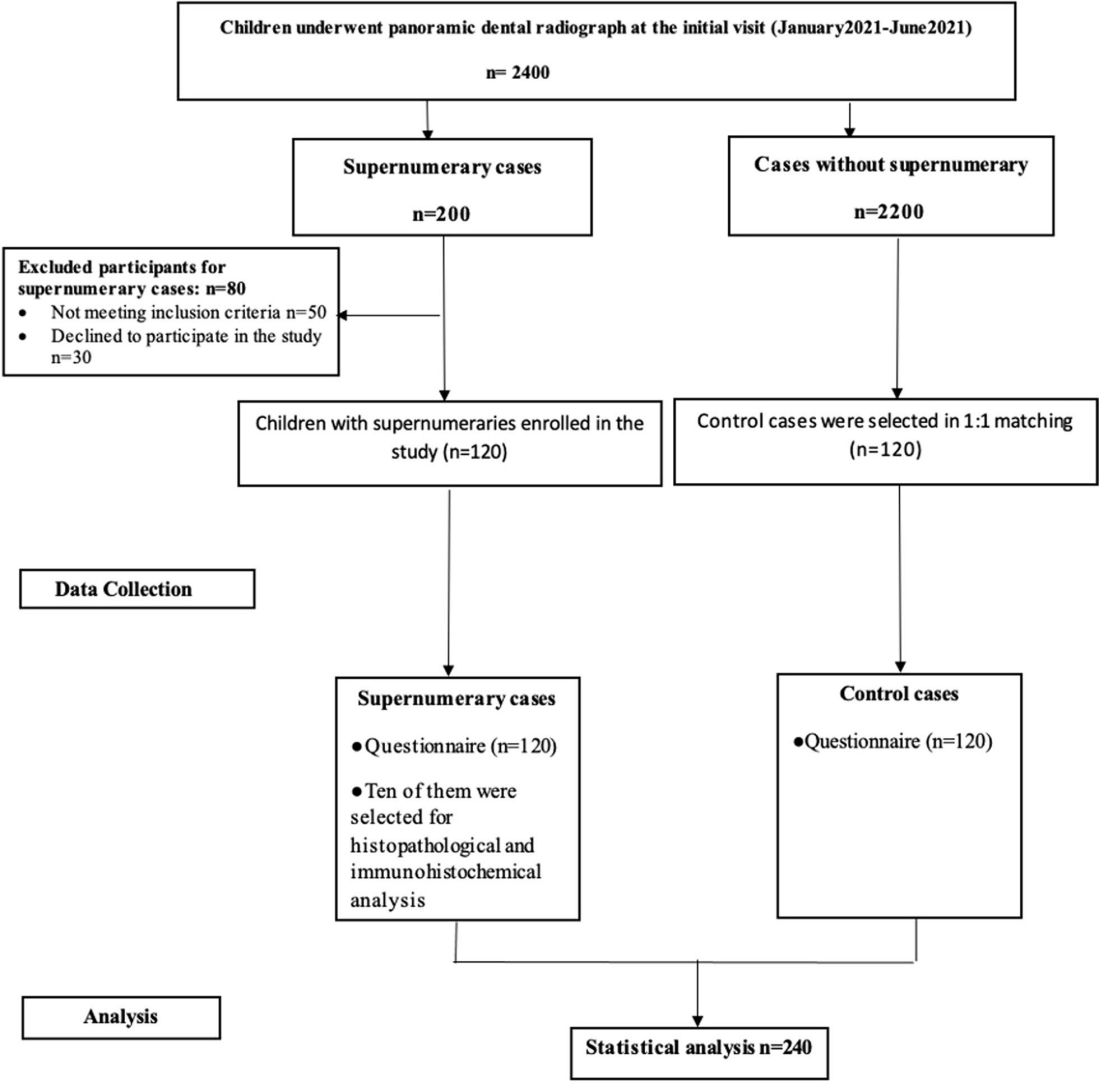
STROBE diagram showing the study protocol

From January 2021 to June 2021, 2400 children attended the Pediatric Dentistry and Dental Public Health Department for whom panoramic radiographs were performed at the initial visits. Two hundred children were presented with supernumerary teeth, where 120 met the inclusion criteria, while 80 were excluded from the study (Fig. 1). The mean age of children with supernumeraries was 8.14 ± 1.779 and 8.22 ± 1.732 in the control group, with an age range between 6 and 11 years, with no statistically significant difference between both groups (*P* = 0.713). No statistically significant difference in gender between the two groups was recorded (*P* = 0.282).

In the test group, males were more frequently affected by supernumeraries (74.2%) than females (25.8%), with a male to female ratio of 2.87:1. The majority of the children had a single supernumerary tooth (64.16%), 33.33% had double supernumeraries, and only 2.5% of the patients had multiple supernumeraries (Table 1).

**Table 1:** Distribution of supernumerary teeth according to gender and number

	Number of Patients with Supernumeraries	Percentage
Gender		
Male	89	74.16
Female	31	25.83
No. of supernumerary teeth		
Single	77	64.16
Double	40	33.33
Multiple	3	2.5
Male patients		
One Supernumerary tooth	52	58.42
≥ 2 Supernumerary teeth	37	41.57
Female patients		
One Supernumerary tooth	25	80.64
≥ 2 Supernumerary teeth	6	19.35

The results of the present study revealed a statistically significant higher number of males with two or more supernumerary teeth (41.57%) compared to female patients (19.35%) (*P* = 0.026).

No significant association was observed between the occurrence of supernumerary teeth and some of the studied risk factors, such as being subjected to dental trauma in the first two years of life, living beside any radiation center, parents’ consanguinity or history of such problems in other relatives (*P* = 0.2416, *P* = 1.00, *P* = 0.3081, and *P* = 1.00, respectively). Moreover, parental information regarding smoking or drugs did not show a statistically significant association to the occurrence of supernumerary teeth compared to controls (*P* = 0.4334, and *P* = 1.00, respectively). However, there was a significant association between supernumerary teeth and the majority of studied risk factors, including maternal feeding practices and drinking mineral water (*P* = 0.0004, *P* = 0.0004, respectively), dental history of severe oral infection or medical history of chemotherapy treatment (*P* = 0.0131, *P* = 0.0131,respectively), using mobile phones or any other electronics (*P* = 0.0054), living beside agricultural fields or industrial areas (*P* < 0.0001, *P* = 0.0004, respectively), maternal illnesses or intake of medications during pregnancy (*P* < 0.0001) as well as positive family history of tumors (*P* < 0.0001) in comparison to controls (Table 2).

**Table 2:** Distribution of studied risk factors between the cases and controls

Questions	Supernumerary cases				Controls				*P* value
	Yes	%	No	%	Yes	%	No	%	
Was the child breast fed?	108	90	12	10	120	100	0	0	0.0004*
Was the drinking water being mineral water?	12	10	108	90	0	0	120	100	0.0004*
Has the child been subjected to any sort of severe infection (oral infections such as gingivitis or abscess) in the first two years of his life?	6	5	114	95	0	0	120	100	0.0131*
Was the child under chemotherapy?	6	5	114	95	0	0	120	100	0.0131*
Has the child been subjected to traumatic Dental Injury (TDI) in the first 2 years?	18	15	102	85	12	10	108	90	0.2416
Did the child play with mobile phones or any other electronics?	18	15	102	85	36	30	84	70	0.0054*
Does the child live besides any center of radiation?	0	0	120	100	0	0	120	100	1.00
Does the child live besides any agriculture fields?	0	0	120	100	30	25	90	75	< 0.0001*
Does the child live besides industrial area?	0	0	120	100	12	10	108	90	0.0004*
Are the mother and father relatives (first-degree consanguine or second-degree consanguine)?	24	20	96	80	18	15	102	85	0.3081
Any other relatives with such problem?	0	0	120	100	0	0	120	100	1.00
Does any of the parents’ smoke?	72	60	48	40	66	55	54	45	0.4334
Does any of the parents take drugs?	0	0	120	100	0	0	120	100	1.00
Was the mother using medications or did she suffer from illness during pregnancy?	30	25	90	75	6	5	114	95	< 0.0001*
Was there a previous history of neoplastic disease in the family?	72	60	48	40	6	5	114	95	< 0.0001*

^*^Statistically significant

**Fig. 2:**
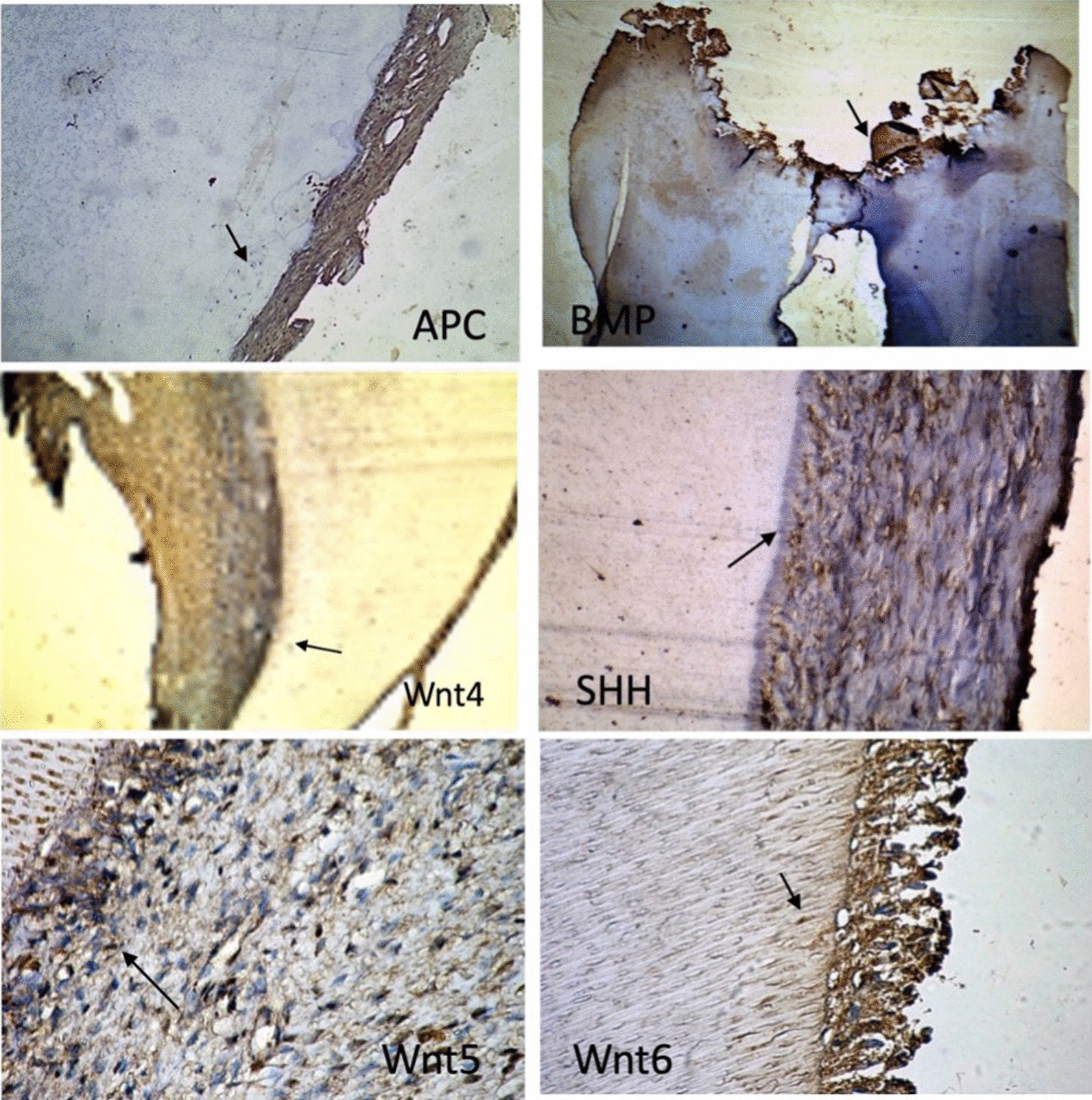
Representative images of the expression levels of Wnt proteins (Wnt 4, Wnt 5 and Wnt 6), sonic hedgehog (SHH) protein, bone morphogenic protein 11 (BMP11) and adenomatous polyposis coli (APC) protein in the studied supernumerary teeth samples. Positive expression is represented as brown immunostaining

**Fig. 3:**
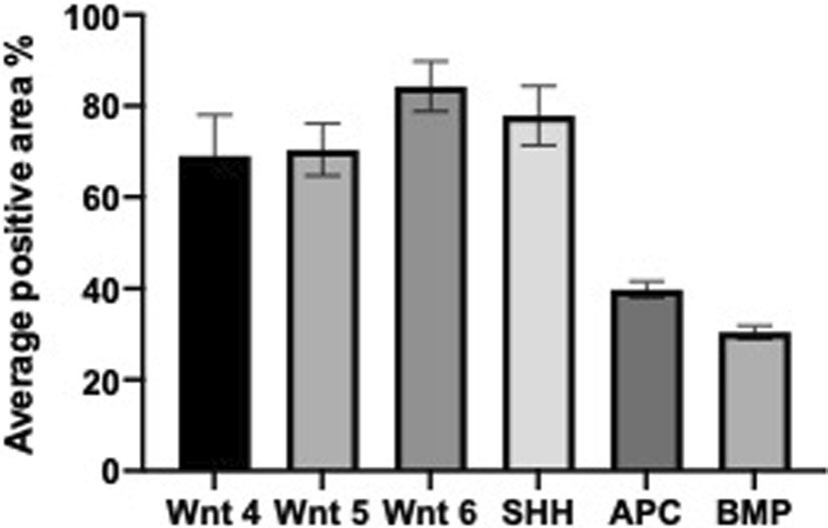
A bar graph showing the average staining intensities of the different proteins

The results of the present study also showed high expression levels of Wnt, SHH and BMP11 proteins and a lower expression of the APC protein in the supernumerary specimens (Fig. 2). The average positive area percentage of Wnt 4 was equal to 69 ± 9.1. Similarly, the average positive area percentage of Wnt 5 and Wnt 6 was also high (70.4 ± 5.7, and 84.3 ± 5.5, respectively). Additionally, the expression of SHH and the BMP11 was enriched with an average positive area percentage of 77.9 ± 6.5 and 39.8 ± 10.8, respectively. In contrast, the APC protein revealed lower expression in the same specimens (30.4 ± 1.48) (Fig. 3).

## Discussion

Despite advances in our knowledge of tooth development and morphogenesis, a better understanding of the possible predisposing factors and molecular pathways involved in supernumerary tooth formation is still needed.

In some cases, with supernumeraries, the excess tooth is left as a replacement to a lost permanent tooth to complete the dentition both functionally and aesthetically [19]. However, in most of the cases included in the present study, the extraction of the supernumerary teeth was required to avoid potential complications.

Results of the present study showed that male patients had a higher number of supernumerary teeth compared to females. A possible association with sex-linked inheritance and an autosomal recessive gene with a lower penetrance rate in females has been theorized as a possible etiology related to the gender variation of supernumerary teeth [20]. This is in agreement with previous reports that showed a gender predominance of males over females in the incidence of supernumerary teeth [3, 21].

A significant association between supernumerary teeth occurrence and most of the studied factors has been reported. This was in agreement with several studies stating that tooth development is a dynamic interaction between genetic and environmental factors. Such factors interact and affect each other, hence environmental factors have been considered as possible etiological factors in the development of supernumerary teeth [7, 22–24]. The significant association between supernumerary teeth occurrence and chemotherapy treatment was in agreement with other studies [25, 26]. They showed that chemotherapy may affect the processes of amelogenesis and dentinogenesis and can lead to abnormalities in the development of tooth buds. The severity of these abnormalities depends on the dosage and frequency of therapeutic cycles, the age of the child at the beginning of oncologic therapy, the nutritional status, and the stage of tooth development [27]. Although there was a statistically significant association between using mobile phones and the occurrence of supernumerary teeth, no clear effect of mobile radiation on teeth has been proved in the literature. However, many countries have recommended measures to minimize mobile radiation exposure and advised moderate use of mobile phones for children [28]. A significant association between supernumerary teeth occurrence and maternal history of illness or taking medications during pregnancy has also been reported. This is in accordance with Garn et al. and Garcia Rincon et al. [29, 30], who stated that environmental factors, including maternal illness, may affect teeth development. Moreover, drugs taken during pregnancy might play a role in increasing the activity of the dental lamina, which is one of the suggested theories in supernumerary teeth formation [6]. According to Vargesson [31], drug intake, like thalidomide, during pregnancy has been recorded as an important cause that might lead to tooth anomalies, including size and number.

Furthermore, the results of the present work showed a significant association between supernumerary teeth existence and positive family history of neoplastic diseases. This could be attributed to the inheritance of the APC deletion, which is known to be responsible for the extra tooth formation through increasing dental lamina activity [11]. Sawai et al. [32] showed an important association between supernumerary teeth and a group of syndromes, including Gardner's syndrome, cleidocranial dysostosis and cleft lip and palate. Some of these syndromes, like Gardner’s syndrome, are associated with multiple adenomatous polyposes and a number of benign and malignant tumors [33].

On the other hand, the results of the present study did not reveal a notable association between the occurrence of supernumerary teeth and having other relatives with such problems, however, other published cases of supernumerary teeth mentioned recurrence within the same family [34, 35]. Additionally, parental smoking was not found to play a role in the existence of supernumerary teeth, nevertheless, Miyake et al. [36] stated that exposure of the pregnant mother to toxic tobacco compounds might alter the expression of genes involved in the development of teeth.

Analysis at the cellular level showed an enrichment of the present samples with Wnt pathway proteins, including Wnt 4, Wnt 5 and Wnt 6. In contrast, a low expression level of the APC protein has been recorded. These results were similar to those reported by Wang et al. [37], who showed deletion of APC or activation of Wnt signaling pathway to play a role in dental epithelium hyperactivity and promote its interaction with adjacent mesenchyme that predispose for extra tooth development [11]. Noteworthy, Gardner's syndrome, a variant of familial adenomatous polyposis, was linked with loss-of-function of germline mutations in the APC gene [38].

Results of the present study also showed that the expression of SHH protein was enriched in the samples. This is in agreement with the fact that SHH signaling is essential for regulating tooth and oral development and human dentition [39]. Moreover, enhanced expression of the BMP11, a member of BMPs belonging to the transforming growth factor (TGF)-beta superfamily, has been noticed in the studied samples. Similarly, Murashima-Suginami et al. [40] and Kiso et al. [41] concluded that BMPs play an essential role in supernumerary tooth formation.

A possible limitation of the present study was that a validation of the results is required using a larger cohort of patients through molecular techniques. Moreover, the morphological type of supernumeraries (supplemental/ conical/ tuberculate) and position (maxillary/mandibular, mesiodens/distomolar) as well as being impacted or erupted have not been investigated. Therefore, further research is required to study these characteristic features and relate them to the possible risk factors affecting the occurrence of supernumerary teeth. Another limitation of the current study was the absence of serially extracted teeth as a control group. However, the expression of the biomarkers of interest has been extensively investigated in normal tooth development in previous studies [42, 43]. It was speculated that the expression would be the same as in supernumerary teeth as they are of normal structure. Within these limitations and based on the results of the present study, several possible risk factors might be involved in supernumerary teeth development. Additionally, the findings of the current work highlighted the importance of the molecular derangement in the development of supernumerary teeth, namely activation of Wnt, SHH and BMP11 pathways and inactivation of the tumor suppressor gene APC. Therefore, supernumerary teeth can be an alarming finding for early diagnosis of neoplasms, highlighting the necessity of regular follow-up of these children.

## Conclusions

The data of the current study suggested that males were more frequently affected by supernumerary teeth than females. Several risk factors were significantly associated with supernumerary teeth. The molecular biomarkers assessment demonstrated a high expression level of pro-tumorigenic proteins such as Wnt and SHH in patients with supernumerary teeth. Further investigations and long-term follow-up studies are required to correlate between supernumerary teeth in childhood and the possible occurrence of neoplasms in adulthood.

## Data Availability

The datasets generated and analyzed during the current study are available in patients’ records attending the Pediatric Dentistry Department, Faculty of Dentistry, Alexandria University, and were used under license for the current study. Restrictions apply to the public availability of these data. However, data from the corresponding author are available upon reasonable request and with the permission of the Pediatric Dentistry Department, Faculty of Dentistry, Alexandria University.

## Abbreviations

Wnt: Wingless
SHH: Sonic hedgehog
APC: Adenomatous polyposis coli
BMP: Bone morphogenic protein
STROBE: Strengthening the Reporting of Observational Studies in Epidemiology
CVI-I: Content validity at the item level
IHC: Immunohistochemistry
SD: Standard deviation
ANOVA: Analysis of Variance
TGF: Transforming growth factor
